# Rifaximin is associated with modest, transient decreases in multiple taxa in the gut microbiota of patients with diarrhoea-predominant irritable bowel syndrome

**DOI:** 10.1080/19490976.2018.1460013

**Published:** 2018-07-18

**Authors:** Anthony A. Fodor, Mark Pimentel, William D. Chey, Anthony Lembo, Pamela L. Golden, Robert J. Israel, Ian M. Carroll

**Affiliations:** a Department of Bioinformatics and Genomics, University of North Carolina at Charlotte, Charlotte, North Carolina, USA;; b Division of Gastroenterology, Cedars-Sinai Medical Center, Los Angeles, California, USA;; c Division of Gastroenterology, Michigan Medicine, Ann Arbor, Michigan, USA;; d Division of Gastroenterology, Beth Israel Deaconess Medical Center, Boston, Massachusetts, USA;; e Nonclinical and Clinical Pharmacology, Clinical and Medical Affairs, Salix Pharmaceuticals, Bridgewater, New Jersey, USA**; f Department of Nutrition and Division of Gastroenterology and Hepatology, University of North Carolina, Chapel Hill, North Carolina, USA

**Keywords:** Antibiotic therapy, colonic microflora, diarrhoea, gastrointestinal immune response, irritable bowel syndrome

## Abstract

Rifaximin, a non-systemic antibiotic, is efficacious for the treatment of diarrhoea-predominant irritable bowel syndrome (IBS-D). Given the emerging association between the gut microbiota and IBS, this study examined potential effects of rifaximin on the gastrointestinal microbial community in patients with IBS-D. TARGET 3 was a randomised, double-blind, placebo-controlled, phase 3 study. Patients with IBS-D initially received open-label rifaximin 550 mg 3 times daily (TID) for 2 weeks. Patients who responded to the initial treatment and then relapsed were randomised to receive 2 repeat courses of rifaximin 550 mg TID or placebo for 2 weeks, with each course separated by 10 weeks. Stool samples were collected at the beginning and end of open-label treatment, at the beginning and end of the first double-blind treatment, and at the end of the study. As a secondary analysis to the TARGET 3 trial, the composition and diversity of the gut microbiota were assessed, from a random subset of patients, using variable 4 hypervariable region 16S ribosomal RNA gene sequencing. Samples from 103 patients were included. After open-label rifaximin treatment for 2 weeks, 7 taxa (e.g. Peptostreptococcaceae, Verrucomicrobiaceae, Enterobacteriaceae) had significantly lower relative abundance at a 10% false discovery rate threshold. The effects of rifaximin were generally short-term, as there was little evidence of significantly different changes in taxa relative abundance at the end of the study (up to 46 weeks) versus baseline. The results suggest that rifaximin has a modest, largely transient effect across a broad range of stool microbes. Future research may determine whether the taxa affected by rifaximin are causally linked to IBS-D.

ClinicalTrials.gov identifier number: NCT01543178.

**Abbreviations**
bp: base pairs
DNA: deoxyribonucleic acid
EOS: end of study
FDR: false discovery rate
GI: gastrointestinal
IBS: irritable bowel syndrome
IBS-D: diarrhoea-predominant irritable bowel syndrome
MDS: multidimensional scaling
OTU: Operational Taxonomic Unit
PCR: polymerase chain reaction
RDP: Ribosomal Data Project
RNA: ribosomal RNA
rRNA: ribosomal RNA
SD: standard deviation
TARGET: Targeted, non-systemic Antibiotic Rifaximin Gut-selective Evaluation of Treatment for IBS-D
TID: 3 times daily
V: clinic visit

## Introduction

Irritable bowel syndrome (IBS) is a functional bowel disorder characterised by recurrent abdominal pain associated with defecation and alterations in bowel habit.^1^ Patients with IBS have recurrent abdominal pain for ≥ 1 day per week during the previous 3 months, on average, and the pain is associated with at least 2 of the following: defecation, changes in stool frequency, changes in stool appearance. Symptoms of IBS may be chronic or episodic.^2^

The pathophysiology of IBS is not entirely clear, but is thought to include various factors: gut-brain interactions, gastrointestinal (GI) sensorimotor dysfunction (e.g. motility, visceral hypersensitivity), alterations in GI permeability, genetics (e.g. polymorphisms, risk loci), immune activity alterations (e.g. cytokine imbalance, increased inflammation), and psychosocial components (e.g. psychiatric conditions).^3-12^ The role of the GI microbial community (gut microbiota) cannot be overlooked, given evidence supporting an association between enteric infections and post-infectious IBS development,^13^^,^ ^14^ alterations in the gut microbiota in patients with IBS versus healthy individuals,^15-18^ and gut microbiota modulation by antibiotics and probiotics improving symptoms of IBS in some patients.^19-21^

Rifaximin, an oral non-systemic GI-targeted antibiotic, has in vitro activity against a variety of gram-negative and gram-positive bacteria.^22-24^ Binding of rifaximin to the β subunit of bacterial DNA-dependent RNA polymerase inhibits transcription and bacterial RNA synthesis.^22^^,^ ^25^ However, the mechanism of action of rifaximin may extend beyond its antimicrobial activity. In 1 study, rifaximin not only increased *Lactobacillus* spp. in the ileum, but also decreased stress-induced mucosal inflammation and normalised visceral hypersensitivity in animal studies.^26^ Further, rifaximin altered bacterial adhesion to epithelial cells in vitro, thus affecting the ability of some bacterial species to colonise the GI tract. The efficacy and safety of rifaximin, approved for the treatment of adults with diarrhoea-predominant IBS (IBS-D), have been demonstrated in various clinical studies.^27-32^ In 2 identically designed, double-blind, phase 3 studies (Targeted, non-systemic Antibiotic Rifaximin Gut-selective Evaluation of Treatment for IBS-D [TARGET] 1 and TARGET 2), a significantly greater percentage of patients randomised to 2-week treatment with rifaximin 550 mg 3 times daily (TID) achieved adequate relief of global IBS symptoms for ≥ 2 of the first 4 weeks post-treatment versus patients randomised to placebo (pooled data, 40.7% vs 31.7%, respectively; p < 0.001).^27^ The TARGET 3 study examined the efficacy and safety of repeat treatment with rifaximin among patients who initially responded (defined as ≥ 30% decrease from baseline in mean weekly pain score and ≥ 50% decrease from baseline in number of days per week with Bristol Stool Scale type 6 or 7 stool for ≥ 2 of the first 4 weeks post-treatment) to 2-week open-label rifaximin 550 mg TID treatment, but relapsed during an 18-week follow-up phase. These patients were randomised to receive 2 repeat courses of treatment with rifaximin 550 mg TID or placebo for 2 weeks. A significantly greater percentage of patients responded to repeat rifaximin treatment versus placebo (38.1% vs 31.5%, respectively; p = 0.03).^32^

Antibiotic resistance develops over a lifetime of antibiotic exposure, but most studies of the gut microbial community have focused on a single exposure to antibiotics with small patient populations. A study that included 21 hospitalised patients receiving β-lactams or fluoroquinolones found a decrease from baseline in the number of microbial taxa of approximately 25% in faecal samples after 7 days, suggesting that composition and structure of the GI microbial community are greatly affected by short-term antibiotic exposure.^33^ Faecal samples from 4 hospitalised patients receiving β-lactams, fluoroquinolones, or lincosamides showed that the composition and diversity of the GI bacterial community fluctuated following antibiotic therapy.^34^ Previous studies^35^^,^ ^36^ reported modest effects of rifaximin treatment on IBS but did not track patients over multiple doses. The current study examined the effects of repeat rifaximin treatment on faecal microbial composition in a large IBS-D population. The goal of this study was to examine the response of the faecal microbial community, as measured by 16S ribosomal RNA (rRNA) sequencing, in a randomly selected subset of patients with IBS-D participating in the TARGET 3 study.

## Patients and Methods

### Study design and patients

The patient population and clinical trial study design have been reported previously.^32^ Briefly, TARGET 3 was a randomised, double-blind, placebo-controlled phase 3 study (ClinicalTrials.gov identifier NCT01543178) of patients aged ≥18 years with a diagnosis of IBS-D (Rome III criteria) whose global IBS symptoms and bloating persisted during a placebo screening phase. Patients were excluded if they were taking probiotics or taking rifaximin or any other antibiotic within 14 days of giving written informed consent. Patients received open-label rifaximin 550 mg TID for 2 weeks, followed by a 4-week treatment-free follow-up to assess response (i.e. improvement in abdominal pain [≥ 30% decrease from baseline in mean weekly pain score] and stool consistency [≥ 50% decrease from baseline in number of days per week with Bristol Stool Scale type 6 or 7 stool] during ≥ 2 of the first 4 weeks post-treatment). Responders continued in the study and were monitored during a treatment-free observation phase to determine IBS symptom recurrence (i.e. loss of treatment response for either weekly abdominal pain or stool consistency for ≥ 3 weeks of a consecutive, rolling 4-week period during the 18-week observation phase). Patients with recurrence were randomised (1:1) in a double-blind manner to 2 repeat treatments of rifaximin 550 mg TID or placebo for 2 weeks, with each course separated by 10 weeks. The protocol conforms to the ethical guidelines of the 1975 Declaration of Helsinki. The protocol was approved by institutional review boards and ethics committees at participating sites, and all patients provided written informed consent. All authors had access to study data and reviewed and approved the final manuscript.

### Sample collection

**Figure 1.**
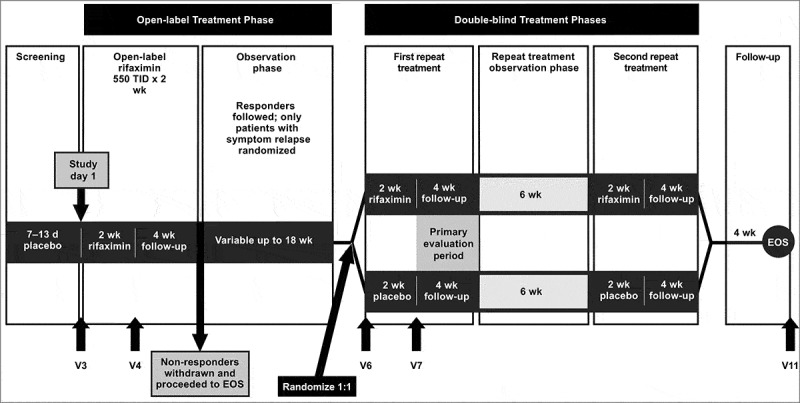
Study design. Adapted with permission from Lembo, et al.^32^ EOS, end of study; TID: 3 times daily, V: clinic visit

All patients participating in TARGET 3 consented to provide stool samples. Patients (responders and non-responders) were randomly selected for inclusion in the substudy using a random number generator. No formal power calculations were conducted to determine sample size. During the planning stages of the current study, there was not a sufficient number of published 16S sequencing studies on antibiotics available to estimate effect size for a formal power analysis. Therefore, the sample size was based on practicality of sequencing costs at the time the study was initiated. Fresh stool samples were collected prospectively from patients at clinic visits (V) before (open-label baseline; V3) and after open-label treatment with rifaximin (open-label week 2; V4), before (double-blind baseline; V6) and after the first double-blind treatment with rifaximin or placebo (double-blind week 2; V7), and at the end of the study (V11; **Figure 1**).^32^ Non-responders to open-label rifaximin were withdrawn from the study after V4. Patients unable to provide a stool sample at clinic visits were offered a stool collection kit for home, with instructions to refrigerate and return the sample to the clinic as soon as possible. Stool samples were separated into 2-mL aliquots in polypropylene cryovials and stored at ≤ –20°C at the clinic site. Samples were shipped on dry ice for long-term storage at ≤ –70°C.

### 16S rRNA sequencing

Frozen stool samples from study participants were shipped to Covance Genomics (Seattle, WA) for genomic sequencing. Samples were thawed at the time of testing and DNA extracted using the QIAamp® DNA Stool Mini Kit (Qiagen, Germantown, MD). Sequencing of the variable 4 hypervariable region of the 16S rRNA gene was performed using the HiSeq 2500 System (Illumina, Inc., San Diego, CA). The sequencing strategy utilised a polymerase chain reaction (PCR) amplicon of 286 base pairs (bp) generated using forward primer F515 (5'-GTGCCAGCMGCCGCGGTAA-3') and reverse primer R806 (5'-GGACTACHVGGGTWTCTAAT-3'). The sequence length on each read was approximately 120 bp from both directions, producing reads that generally did not overlap. Moreover, the strategy utilised an initial PCR reaction followed by ligation of the Illumina primers. Therefore, the 5'- orientation of the DNA sequences in the forward and reverse reads are mixed in the dataset.

The full dataset consisted of 675 faecal samples (n = 103), which generated 2,309,172,633 paired-end 16S rRNA sequence reads following high-throughput sequencing. A total of 1,868,592,999 reads (∼ 81% of all sequenced paired ends) were called version 2.6 of the Ribosomal Database Project (RDP) algorithm to family at the 50% confidence level, with an identical call on both non-overlapping paired ends, and were included for downstream analysis. A total of 666 stool samples representing 440 unique combinations of patient and time point were included in the analysis (**Table 1**). In the case of duplicate samples for the same combination of patient and time point, the sample with the highest total number of sequences was used (**Supplementary Table S1**). All sequences are available under the Sequence Read Archive BioProject ID PRJNA391915 (http://www.ncbi.nlm.nih.gov/bioproject/391915). Please also see online supplementary materials for additional information and analyses (Supplementary Tables S1-S7 and Figures S1-S5).

**Table 1.** Number of samples sequenced from stool samples submitted at each visit

Visit	Samples, n*^a^*	Mean ± SD number of sequences called to family
Open-label baseline (V3)	101	3,465,091 ± 2,469,103
Open-label week 2 (V4)	102*^b^*	3,511,837 ± 2,496,318
Double-blind baseline (V6)	69	3,177,231 ± 2,158,671
Double-blind week 2 (V7)	72	4,199,975 ± 3,653,364
End of study (V11)	96	4,095,411 ± 2,361,115

A total of 103 patients with IBS-D were included in the study, but no patients had samples evaluated at all time points.

b Individual patients could have had ≥ 1 sample evaluated at a given time point.

IBS-D: diarrhoea-predominant irritable bowel syndrome; SD: standard deviation; V: clinic visit

### Data analysis

The analysis pipeline used the RDP algorithm applied to each paired read with a threshold of 50%.^37^ RDP calls were used at the family level because at these read lengths, the RDP algorithm is not generally reliable down to genus. Sequences were included for analysis when the RDP algorithm yielded an identical call on both non-overlapping paired end reads. The analysis strategy had several advantages: (1) it guarded against low sequence quality, chimeras, and non-microbial contamination because if there was a high rate of error from these sources, one would not expect the RDP algorithm to consistently agree for both non-overlapping paired ends; (2) it included only taxa on which a consistent call is generated from the RDP algorithm for both paired-end sequences despite 16S reads that generally do not overlap; the results, therefore, would be robust and not dependent on sequencing a particular sub-region of the variable 4 region; (3) it was computationally attractive despite the large number of sequences generated because the RDP algorithm is both fast and easy to make parallel; and (4) it was insensitive to the fact that orientations in both directions were present in the sequences.

The non-overlapping reads generated by the pipeline meant that there were not long continuous stretches of the 16S rRNA gene available. In general, algorithms have not been developed to perform detailed phylogenetic analyses on non-continuous 16S rRNA sequences in both orientations. Therefore, the analysis was restricted to reads in which both the forward and backwards reads agreed. No other quality score filtering was performed. Because quality score filtering can be described as a “compromise between quantitative accuracy and error rates,”^38^ and the taxa impacted by rifaximin were generally low in relative abundance, the existence of similar classification on non-overlapping reads was deemed to be a sufficient guard against sequencing error. Below the family level, agreement between the 2 reads decreased: there were ∼ 18% fewer assignments in which the 2 reads agreed at the genus level when compared with the family level. Therefore, analyses are reported in this manuscript at the family level, although an analysis at the genus level yielded substantially similar results, with 25 taxa different between the V3 and V4 time points at a 10% false discovery rate (FDR; **Supplementary Table S2**). Alternative analysis paths with different normalisation schemes and utilising DADA 2 to generate de novo clusters are described in the Supplementary Material.

Data included in the diversity index were composed of measures of richness (number of families in each sample corrected for different samples having different numbers of sequences), evenness (calculated by Shannon's equitability, with values ranging from 0 to 1 [complete evenness]), and Shannon diversity (measure of overall community complexity).

The equation used for Shannon diversity was:

$$H=\;-\sum_{i=1}^{S}P_{i}\;\times \ln P_{i}$$

where *H* = Shannon diversity index; P*_i_* = fraction of the entire population composed of species *i* (proportion of a species *i* relative to the total number of species present, not encountered); and *S* = the number of species encountered.

The equation used for Shannon evenness or equitability was:

$$E_{H}=\frac{H}{H_{max}}=\frac{H}{\ln S}\;$$

where *E_H_* = Shannon equitability or evenness.

For richness, each sample was rarified (sampled without replacement) to 3839 sequences (number of sequences from the sample with the fewest matched paired ends to family), and the number of distinct families seen in each rarified sample was calculated. Richness is reported as the average of 20 such rarefaction calculations.

### Statistical analysis

The RDP counts to family level were log-normalised as described previously.^39^ Count tables were normalised as follows:

$$\log_{10}\;\left(\frac{Bacteria\;count\;for\;sample\;i}{Number\;of\;sequences\;in\;sample\;i}*Average\;\#of\;sequences\;per\;sample+1\right)$$

This equation minimises differences in the impact of adding the pseudo-count of 1 to each sample. Data were log-transformed with this formula prior to all multidimensional scaling (MDS) ordination and statistical tests, except for the Wilcoxon tests shown in **Supplementary Table S3** and the rarified DADA 2 pipeline shown in **Supplementary Table S4 and Figure S4**. Operational taxonomic units (OTUs) absent in more than 10% of the samples were discarded.

Taxa relative abundances represent the log_10_ sequenced count for each sample, normalised so that the total number of sequence counts is identical for all samples. To preserve power in corrections for multiple hypothesis testing, we built statistical models only for taxa that were present in ≥ 10% of samples. Correction for multiple hypothesis testing was performed with the Benjamini–Hochberg procedure using the p.adjust function in R. MDS ordination was performed using the capscale function in the Vegan package in R (Oksanen *et al*, The R Foundation, Vienna, Austria) with Bray-Curtis distance. Mixed linear models, described in the Supplementary Materials, were created using the gls method in R.

Including the 96 stool samples from the pilot study, 440 unique combinations of patients and time points were sequenced (**Table**). Some stool samples were selected for multiple sequencing reactions at a specific visit (e.g. resequencing for insufficient sequence depth, technical replicates); in these cases, the sequencing data with the greatest sequencing depth were taken.

### Reproducibility and Batch Effects

**Figure 2.**
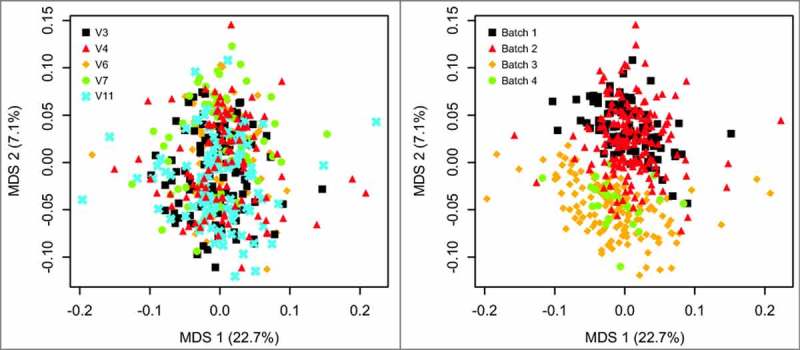
Multidimensional scaling (MDS) ordination at the family level for all samples colored by visit (left panel) and batch (right panel). Batch 1 and batch 2 show substantial overlap, as do batch 3 and batch 4. Tests from 9999 ADONIS permutations yielded a significant difference (p = 0.016) for V3 vs V4 and the effect of patient (p < 0.0001), but a non-significant difference between V3 and V11 (p = 0.0752).

Reproducibility may affect the use of microbial sequence data in a clinical setting. Reproducibility was demonstrated within a batch across extraction, PCR, and sequencing reactions for all but the most rare taxa (**Supplementary text, Supplementary Figure S2**). Due to the high concordance between these initial sequencing runs, these runs are considered to represent a single batch (hereafter referred to as batch 1). The entire dataset was generated over four distinct batches (run at the sequencer at different times) and did show evidence of a batch effect with an MDS ordination, based on family showing similarity between the first two batches of sequencing runs in comparison to the last two batches of sequencing runs **(****Figure 2**).

Y= time  point + subjectID

## Results

### Effects of open-label rifaximin treatment on gut microbiota

A total of 103 patients were randomly selected from the larger TARGET 3 cohort for inclusion in the stool microbiota analysis, of which 37 and 36 patients received double-blind rifaximin and placebo, respectively; 30 patients received open-label rifaximin only. Most of the 103 patients were white (82.5%) and female (73.8%), with a median age of 48.0 years (range, 19–85 years). Demographic characteristics were comparable between double-blind groups.

**Figure 3.**
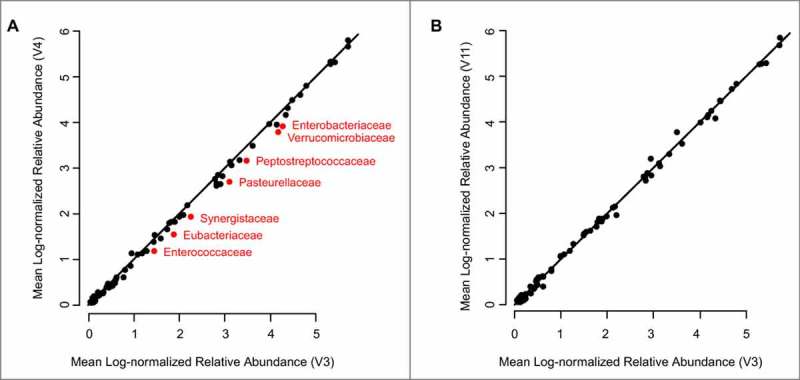
Rifaximin induces a small, transient reduction in the relative abundance of multiple taxa. (A) For 74 non‒rare taxa observed in ≥10% of all samples, the mean relative abundance of each taxon is shown in V3 (n = 101) and V4 (n = 102) samples. (B) For the same taxa, the mean relative abundance of taxa is shown in V3 and V11. The symbols in red indicate taxa that were significantly different in paired Wilcoxon test comparisons of log-transformed data (**Supplementary Table S3**). The black line is the identity line (V3 = V4). V: clinic visit

Overall, the microbial community was stable following rifaximin treatment. **Figure 3****A** compares the average relative abundance for 74 non-rare taxa (observed in ≥ 10% of all samples) at the family level at V3 (x-axis) and V4 (y-axis) across 101 patients sampled for V3 and V4 during the open-label phase. While the relative abundance of each family was similar before and after open-label rifaximin treatment, many of the taxa were slightly below the identity line, indicating some depression of relative abundance associated with rifaximin treatment. In addition to examining each taxon individually, summary statistics for each sample that reflect overall microbial community complexity (diversity) and the number of taxa in each sample (richness) were examined. A slight decrease in Shannon diversity and richness associated with rifaximin treatment were observed (**Supplementary Figure S1; Supplementary Table S3**), suggesting that rifaximin treatment made the microbial community a bit less complex. By contrast, in comparing time points V3 with V11 (**Figure 3****B**), there was no evidence of systematic depression associated with rifaximin treatment, indicating the effects of rifaximin treatment on the microbial community were largely reversed at the end of the study.

A non-parametric paired Wilcoxon test was conducted to compare V3 and V4 time points (Supplementary Tables S4-S7). At a 10% FDR cutoff, 7 taxa plus Shannon diversity and richness differed significantly between V3 and V4 (marked symbols in **Figure 3****A**; p values online in **Supplementary Table S6**). All significant taxa were lower following rifaximin treatment, consistent with rifaximin having a small effect on depressing multiple microbial taxa. However, the effects of rifaximin were highly modest; none of the significantly different taxa changed by more than 2.5-fold between V3 and V4 time points (**Supplementary Table S4**), and no effect size of the association with rifaximin treatment was greater than 4% (**Supplementary Table S5**). An alternative analysis path, based on DADA 2 clustering on rarefied data (**Figure S4**; methods described in the Supplementary Material), failed to find any significant OTU differences between the V3 and V4 time points, perhaps reflecting the possible loss of power that is common when rarefication has been utilised.^40^ Moreover, the choice of log-transforming the data prior to performing the paired Wilcoxon test also modestly impacts the data; performing the paired test on log-transformed data yields 7 significantly different taxa between V3 and V4 (**Figure 3****A**; **Supplementary Table S6**), while performing the test on non-log transformed data yields an additional 4 significant taxa (**Supplementary Table S3**). These results demonstrate that the findings of an association between rifaximin and the microbial community are small enough that their statistical significance is dependent on the exact analysis path chosen. These findings of the dependence of statistical significance on details of the analysis path emphasise the modest effect that rifaximin treatment has on the microbial community when compared with the stronger tendency of the microbial community to be stable over time within individuals. No significant changes were seen by the paired Wilcoxon test between the V3 and V11 (end of study) timepoints, indicating that the effects of rifaximin were transient.

In this study, samples were sequenced in 4 batches; MDS ordination shows some evidence of a batch effect (**Figure 3**). However, a similar pattern of taxa depressed by rifaximin was shown in the 51 patients whose V3 and V4 samples were sequences in the first 2 batches when compared with the distinct set of 41 patients whose samples were sequenced in batches 3 and 4 (**Supplementary Figure S5**). Our results, therefore, cannot easily be explained only by differences caused by different batches of sequences.

**Figure 4.**
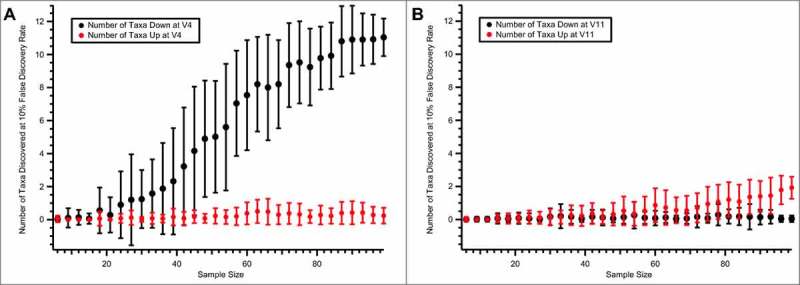
The number of taxa observed as different from V3 to V4 (A) and to V11 (B) using smaller sample sizes. At each sample size, the number of patients indicated on the x-axis was subsampled 50 times (without replacement). The mean and SD of the number of taxa that were detected at a 10% FDR by the paired *t*-test are shown for taxa with relative abundances that were depressed (black) or enhanced (red) in association with rifaximin treatment. FDR: false discovery rate; SD: standard deviation; V: clinic visit

The sample size (∼ 100 patients) is unusually large for a study of antibiotic effects on the microbial community. Simulations were conducted in which different numbers of patients were subsampled (without replacement) and compared at V3 and V4. In these simulations, to maximise the power with small sample sizes, a paired *t*-test instead of the paired Wilcoxon test was used. At each sample size, the paired *t*-test was repeated 50 times to determine the number of taxa depressed (**Figure 4****A,** black) or enhanced (**Figure 4****A**, red) with rifaximin treatment. These simulations revealed the likelihood that no differences between V3 and V4 would be observed with a sample size < 20 patients. Even with ∼ 40 patients, some of the 50 permutations revealed no significant differences. The number of taxa that are depressed in association with rifaximin treatment appeared to saturate at ∼ 85 patients. There were, however, apparent plateaus between 60 and 65 patients and between 70 and 80 patients, but additional taxa were still found at the larger sample size. One cannot, therefore, be certain that if substantially more patients were included, some of the taxa that trended downward (**Figure 3****A**) might not have been found significant at a 10% FDR threshold. By contrast, there appeared to be, on average, ∼1 taxon that was significantly increased at the end of the study across permutations, although nearly the entire cohort of ∼ 90 to 95 patients was needed to reliably observe at least one significantly changed taxon, indicating that the effect size associated with this enhancement was modest.

### Effects of double-blind rifaximin treatment on gut microbiota

**Figure 5.**
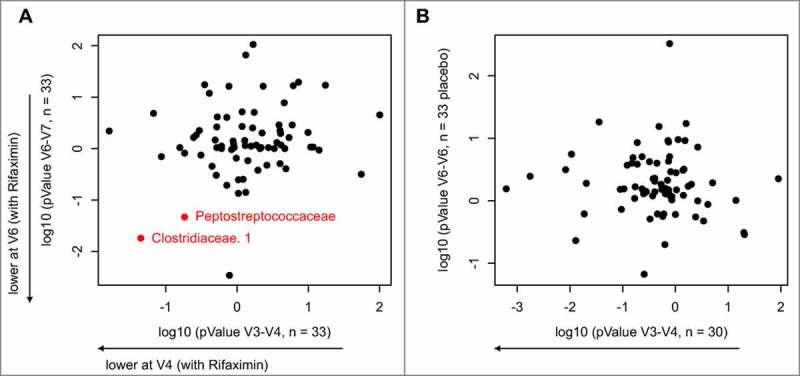
Comparison between the first (V3–V4) and second (V6–V7) treatment of uncorrected p values for non‒rare taxa for the null hypothesis that patients responded to rifaximin (A) or placebo (B) as evaluated by the paired t-test. The y-axis shows the p value for the null hypothesis that treatment had no effect for each taxa for the V6–V7 time point (second treatment) where patients were treated at this time point with either rifaximin (A) or placebo (B). The x-axis shows the results of evaluation of the null hypothesis for the V3 and V4 time points (first treatment), where all patients were treated with rifaximin. To distinguish the direction of response of each treatment, if the mean after treatment was higher than before treatment, the log p value was multiplied by –1. Only patients for whom the researchers had samples for all 4 time points were included in this analysis (33 patients treated with rifaximin for V6–V7; 30 patients in the placebo group for V6–V7) V: clinic visit

In the double-blind phase, responders to open-label rifaximin treatment were randomised to receive either rifaximin or placebo. Paired stool samples were obtained at V6 and V7 from patients receiving rifaximin and placebo (34 patients each). Given our power estimates (**Figure 4**), the expectation might be that this sample size may not have significant differences between rifaximin and placebo; indeed, no significant differences were observed between V6 and V7 time points for rifaximin at a 10% FDR (**Supplementary Table S5**). However, at a simple uncorrected threshold value of p < 0.05, 7 taxa differed significantly between V6 and V7, including *Peptostreptococcaceae* and *Clostridiaceae*. If an analysis is generated that is constrained to only the 33 patients who received 2 double-blind rifaximin treatments and for which we have sequences for both V3–V4 and V6–V7 time points (**Figure 5****A**), it is noted that the p values are not substantially smaller for the second rifaximin treatment and there is one taxa (Clostridiaceae.1), that is significant at the uncorrected threshold of p < 0.05 for both time points. As may be expected, there is very little agreement between changes in the V3–V4 rifaximin phase and the V6–V7 placebo phase in a similar analysis performed on the 30 patients in the placebo group, for which there are sequences for the V3, V4, V6, and V7 time points (**Figure 5****B**). While limited by the small sample size in the double-blind phase, these results are consistent with a model in which there is not a large increase in effect size during repeat rifaximin treatment in the double-blind phase.

## DISCUSSION

The findings of this study suggest that retreatment with rifaximin has a modest, transient effect on the faecal microbial community, an observation consistent with previous studies that found modest changes in community composition in response to rifaximin.^35^^,^ ^36^ This study is unique in that it is one of the largest to demonstrate reproducibility within a single pipeline, crucial for the clinical use of enteric microbial data. The power simulations suggest that some changes observed in bacterial taxa in response to rifaximin over time with a large patient population would be missed with a smaller sample size or less sequencing depth. Based on the power simulation, there would be little value in measuring the microbial community for rifaximin in clinical trials with ∼ 70 patients or fewer. This study is the first to measure the response of the microbial community to rifaximin over multiple treatments. Data were consistent across the 2 study phases, with rifaximin open-label treatment and double-blind retreatment demonstrating approximately the same magnitude of effect on the faecal microbial community, suggesting that multiple treatments may not enhance the modest effect of rifaximin on the microbial community, although the sample size for V6 and V7 may limit the power to detect changes during the retreatment phase. Finally, results indicate that time alone had no apparent effect on the faecal microbial community as comparisons of V3 versus V11 and V6 versus V7 time points were mostly negative.

Findings of this comprehensive study indicate little evidence of sustained changes to faecal microbiota following short-term rifaximin retreatment. The finding that no taxa remained significantly depressed at V11 is consistent with the concept that rifaximin modulates multiple microbial taxa, albeit transiently.^41^ The changes observed with rifaximin treatment were modest, and the statistical significance of these changes was dependent on the analysis path chosen.

One potential limitation of this study is the use of stool samples, which are composed of layers of distinct microbial communities.^42^ In contrast, mucosal biopsies are representative of a single microbial community.^43^ Further, stool samples from patients with IBS-D have an altered faecal structure (e.g. increased mucus, which forms mucus septa or striae) versus healthy individuals with firmer stools.^42^ Also, sequencing of the 16S rRNA gene provides information regarding the specific bacteria that comprise the faecal microbial community.^44^ However, other DNA sequencing-based metagenomic approaches, such as whole-genome shotgun sequencing, are not dependent on sequencing of specific DNA regions, but randomly sequence all fragmented DNA from the sample. Future studies that utilize this method will allow for functional assignments of gene families in the gut microbial community, as well as the potential for strain-level taxonomic resolution.^45^ Lastly, the findings of this study were limited to rifaximin use in patients with IBS-D, and lack of a non-IBS control group limits the ability to determine which members of the microbial community are associated with IBS symptoms. Future studies that mechanistically link individual taxa directly to IBS may allow for evaluation of the hypothesis that the taxa that change in response to rifaximin treatment are the cause of rifaximin's beneficial effects.

## CONCLUSIONS

In conclusion, rifaximin retreatment has only a modest, transient effect on the faecal microbial community. However, the impact on a few select taxa suggests that modulation of these taxa may be part of the mechanism of action of rifaximin. Future studies in animal models are needed to examine this hypothesis. Furthermore, future studies are warranted to explore the ability of the gut microbiome composition to predict response to rifaximin treatment.

## Disclosure of potential conflicts of interest

These authors disclose the following: Anthony A. Fodor has served as a consultant for Salix Pharmaceuticals. Mark Pimentel has served as a consultant for and has received research funding from Salix Pharmaceuticals. In addition, Cedars-Sinai Medical Center has a licensing agreement with Salix Pharmaceuticals. William D. Chey has served as a consultant for Salix Pharmaceuticals. Anthony Lembo has served as a consultant for Salix Pharmaceuticals. Pamela L. Golden is a former employee of Salix Pharmaceuticals. Robert J. Israel is an employee of Salix Pharmaceuticals or its affiliates. Ian M. Carroll has served as a consultant for Salix Pharmaceuticals.

## References

[1] Lacy BE, Mearin F, Chang L, Chey WD, Lembo AJ, Simren M, Spiller R Bowel disorders. Gastroenterology 2016; 150:1393–407. 10.1053/j.gastro.2016.02.031 27144627

[2] Chey WD, Kurlander J, Eswaran S Irritable bowel syndrome: a clinical review. JAMA 2015; 313:949–58. 25734736 10.1001/jama.2015.0954

[3] Knight JR, Locke GR III, Zinsmeister AR, Schleck CD, Talley NJ Family history of mental illness or alcohol abuse and the irritable bowel syndrome. J Psychosom Res 2015; 78:237–41. 25582802 10.1016/j.jpsychores.2014.11.021 PMC4505618

[4] Bashashati M, Rezaei N, Shafieyoun A, McKernan DP, Chang L, Ohman L, Quigley EM, Schmulson M, Sharkey KA, Simren M Cytokine imbalance in irritable bowel syndrome: a systematic review and meta-analysis. Neurogastroenterol Motil 2014; 26:1036–48. 24796536 10.1111/nmo.12358

[5] Rana SV, Sharma S, Sinha SK, Parsad KK, Malik A, Singh K Pro-inflammatory and anti-inflammatory cytokine response in diarrhoea-predominant irritable bowel syndrome patients. Trop Gastroenterol 2012; 33:251–56. 23923350 10.7869/tg.2012.66

[6] Guilarte M, Santos J, de Torres I, Alonso C, Vicario M, Ramos L, Martinez C, Casellas F, Saperas E, Malagelada JR Diarrhoea-predominant IBS patients show mast cell activation and hyperplasia in the jejunum. Gut 2007; 56:203–09. 17005763 10.1136/gut.2006.100594 PMC1856785

[7] Vivinus-Nébot M, Frin-Mathy G, Bzioueche H, Dainese R, Bernard G, Anty R, Filippi J, Saint-Paul MC, Tulic MK, Verhasselt V, et al. Functional bowel symptoms in quiescent inflammatory bowel diseases: role of epithelial barrier disruption and low-grade inflammation. Gut 2014; 63:744–52. 23878165 10.1136/gutjnl-2012-304066

[8] Ahn JY, Lee KH, Choi CH, Kim JW, Lee HW, Kim JW, Kim MK, Kwon GY, Han S, Kim SE, et al. Colonic mucosal immune activity in irritable bowel syndrome: comparison with healthy controls and patients with ulcerative colitis. Dig Dis Sci 2014; 59:1001–11. 24282051 10.1007/s10620-013-2930-4

[9] Holliday EG, Attia J, Hancock S, Koloski N, McEvoy M, Peel R, D'Amato M, Agreus L, Nyhlin H, Andreasson A, et al. Genome-wide association study identifies two novel genomic regions in irritable bowel syndrome. Am J Gastroenterol 2014; 109:770–72. 24797007 10.1038/ajg.2014.56

[10] Wouters MM, Lambrechts D, Knapp M, Cleynen I, Whorwell P, Agreus L, Dlugosz A, Schmidt PT, Halfvarson J, Simren M, et al. Genetic variants in *CDC42* and *NXPH1* as susceptibility factors for constipation and diarrhoea predominant irritable bowel syndrome. Gut 2014; 63:1103–11. 24041540 10.1136/gutjnl-2013-304570

[11] Zhou Q, Zhang B, Verne GN Intestinal membrane permeability and hypersensitivity in the irritable bowel syndrome. Pain 2009; 146:41–46. 19595511 10.1016/j.pain.2009.06.017 PMC2763174

[12] Shulman RJ, Jarrett ME, Cain KC, Broussard EK, Heitkemper MM Associations among gut permeability, inflammatory markers, and symptoms in patients with irritable bowel syndrome. J Gastroenterol 2014; 49:1467–76. 24435814 10.1007/s00535-013-0919-6 PMC4102674

[13] Zanini B, Ricci C, Bandera F, Caselani F, Magni A, Laronga AM, Lanzini A Incidence of post-infectious irritable bowel syndrome and functional intestinal disorders following a water-borne viral gastroenteritis outbreak. Am J Gastroenterol 2012; 107:891–99. 22525306 10.1038/ajg.2012.102

[14] Nair P, Okhuysen PC, Jiang ZD, Carlin LG, Belkind-Gerson J, Flores J, Paredes M, DuPont HL Persistent abdominal symptoms in US adults after short-term stay in Mexico. J Travel Med 2014; 21:153–58. 24621006 10.1111/jtm.12114

[15] Durbán A, Abellán JJ, Jimenéz-Hernández N, Salgado P, Ponce M, Ponce J, Garrigues V, Latorre A, Moya A Structural alterations of faecal and mucosa-associated bacterial communities in irritable bowel syndrome. Environ Microbiol Rep 2012; 4:242–47. 23757279 10.1111/j.1758-2229.2012.00327.x

[16] Durbán A, Abellán JJ, Jiménez-Hernández N, Artacho A, Garrigues V, Ortiz V, Ponce J, Latorre A, Moya A Instability of the faecal microbiota in diarrhoea-predominant irritable bowel syndrome. FEMS Microbiol Ecol 2013; 86:581–89. 23889283 10.1111/1574-6941.12184

[17] Carroll IM, Ringel-Kulka T, Keku TO, Chang YH, Packey CD, Sartor RB, Ringel Y Molecular analysis of the luminal- and mucosal-associated intestinal microbiota in diarrhea-predominant irritable bowel syndrome. Am J Physiol Gastrointest Liver Physiol 2011; 301:G799–G807. 21737778 10.1152/ajpgi.00154.2011 PMC3220325

[18] Codling C, O'Mahony L, Shanahan F, Quigley EMM, Marchesi JR A molecular analysis of fecal and mucosal bacterial communities in irritable bowel syndrome. Dig Dis Sci 2010; 55:392–97. 19693670 10.1007/s10620-009-0934-x

[19] Hungin APS, Mulligan C, Pot B, Whorwell P, Agreus L, Fracasso P, Lionis C, Mendive J, Philippart de Foy JM, Rubin G, et al. Systematic review: probiotics in the management of lower gastrointestinal symptoms in clinical practice – an evidence-based international guide. Aliment Pharmacol Ther 2013; 38:864–86. 23981066 10.1111/apt.12460 PMC3925990

[20] Frissora CL, Cash BD Review article: the role of antibiotics vs. conventional pharmacotherapy in treating symptoms of irritable bowel syndrome. Aliment Pharmacol Ther 2007; 25:1271–81. 17509095 10.1111/j.1365-2036.2007.03313.x

[21] Pimentel M, Chatterjee S, Chow EJ, Park S, Kong Y Neomycin improves constipation-predominant irritable bowel syndrome in a fashion that is dependent on the presence of methane gas: subanalysis of a double-blind randomized controlled study. Dig Dis Sci 2006; 51:1297–301. 16832617 10.1007/s10620-006-9104-6

[22] Jiang ZD, DuPont HL Rifaximin: in vitro and in vivo antibacterial activity–a review. Chemotherapy 2005; 51:67–72. 15855749 10.1159/000081991

[23] Gillis JC, Brogden RN Rifaximin. A review of its antibacterial activity, pharmacokinetic properties and therapeutic potential in conditions mediated by gastrointestinal bacteria. Drugs 1995; 49:467–84. 7774516 10.2165/00003495-199549030-00009

[24] Marchese A, Salerno A, Pesce A, Debbia EA, Schito GC In vitro activity of rifaximin, metronidazole and vancomycin against *Clostridium difficile* and the rate of selection of spontaneously resistant mutants against representative anaerobic and aerobic bacteria, including ammonia-producing species. Chemotherapy 2000; 46:253–66. 10859431 10.1159/000007297

[25] Xifaxan(rifaximin) tablets, for oral use [package insert]. Bridgewater, NJ: Salix Pharmaceuticals; 2015.

[26] Xu D, Gao J, Gillilland M III, Wu X, Song I, Kao JY, Owyang C Rifaximin alters intestinal bacteria and prevents stress-induced gut inflammation and visceral hyperalgesia in rats. Gastroenterology 2014; 146:484–96. 24161699 10.1053/j.gastro.2013.10.026 PMC3939606

[27] Pimentel M, Lembo A, Chey WD, Zakko S, Ringel Y, Yu J, Mareya SM, Shaw AL, Bortey E, Forbes WP Rifaximin therapy for patients with irritable bowel syndrome without constipation. N Engl J Med 2011; 364:22–32. 21208106 10.1056/NEJMoa1004409

[28] Pimentel M, Park S, Mirocha J, Kane SV, Kong Y The effect of a nonabsorbed oral antibiotic (rifaximin) on the symptoms of the irritable bowel syndrome: a randomized trial. Ann Intern Med 2006; 145:557–63. 17043337 10.7326/0003-4819-145-8-200610170-00004

[29] Meyrat P, Safroneeva E, Schoepfer AM Rifaximin treatment for the irritable bowel syndrome with a positive lactulose hydrogen breath test improves symptoms for at least 3 months. Aliment Pharmacol Ther 2012; 36:1084–93. 23066911 10.1111/apt.12087

[30] Pimentel M, Morales W, Chua K, Barlow G, Weitsman S, Kim G, Amichai MM, Pokkunuri V, Rook E, Mathur R, et al. Effects of rifaximin treatment and retreatment in nonconstipated IBS subjects. Dig Dis Sci 2011; 56:2067–72. 21559740 10.1007/s10620-011-1728-5

[31] Schoenfeld P, Pimentel M, Chang L, Lembo A, Chey WD, Yu J, Paterson C, Bortey E, Forbes WP Safety and tolerability of rifaximin for the treatment of irritable bowel syndrome without constipation: a pooled analysis of randomised, double-blind, placebo-controlled trials. Aliment Pharmacol Ther 2014; 39:1161–68. 24697851 10.1111/apt.12735 PMC4112801

[32] Lembo A, Pimentel M, Rao SS, Schoenfeld P, Cash B, Weinstock LB, Paterson C, Bortey E, Forbes WP Repeat treatment with rifaximin is safe and effective in patients with diarrhea-predominant irritable bowel syndrome. Gastroenterology 2016; 151:1113–21. 27528177 10.1053/j.gastro.2016.08.003

[33] Panda S, El khader I, Casellas F, Lopez VJ, Garcia CM, Santiago A, Cuenca S, Guarner F, Manichanh C Short-term effect of antibiotics on human gut microbiota. PLoS One 2014; 9:e95476. 24748167 10.1371/journal.pone.0095476 PMC3991704

[34] Perez-Cobas AE, Artacho A, Knecht H, Ferrus ML, Friedrichs A, Ott SJ, Moya A, Latorre A, Gosalbes MJ Differential effects of antibiotic therapy on the structure and function of human gut microbiota. PLoS One 2013; 8:e80201. 24282523 10.1371/journal.pone.0080201 PMC3839934

[35] Acosta A, Camilleri M, Shin A, Linker Nord S, O'Neill J, Gray AV, Lueke AJ, Donato LJ, Burton DD, Szarka LA, et al. Effects of rifaximin on transit, permeability, fecal microbiome, and organic acid excretion in irritable bowel syndrome. Clin Transl Gastroenterol 2016; 7:e173. 27228404 10.1038/ctg.2016.32 PMC4893683

[36] Zeber-Lubecka N, Kulecka M, Ambrozkiewicz F, Paziewska A, Goryca K, Karczmarski J, Rubel T, Wojtowicz W, Mlynarz P, Marczak L, et al. Limited prolonged effects of rifaximin treatment on irritable bowel syndrome-related differences in the fecal microbiome and metabolome. Gut Microbes 2016; 7:397–413. 27662586 10.1080/19490976.2016.1215805 PMC5046165

[37] Wang Q, Garrity GM, Tiedje JM, Cole JR Naive Bayesian classifier for rapid assignment of rRNA sequences into the new bacterial taxonomy. Appl Environ Microbiol 2007; 73:5261–67. 17586664 10.1128/AEM.00062-07 PMC1950982

[38] Tourlousse DM, Yoshiike S, Ohashi A, Matsukura S, Noda N, Sekiguchi Y Synthetic spike-in standards for high-throughput 16S rRNA gene amplicon sequencing. Nucleic Acids Res 2017; 45:e23. 27980100 10.1093/nar/gkw984 PMC5389483

[39] Sanapareddy N, Legge RM, Jovov B, McCoy A, Burcal L, Araujo-Perez F, Randall TA, Galanko J, Benson A, Sandler RS, et al. Increased rectal microbial richness is associated with the presence of colorectal adenomas in humans. ISME J 2012; 6:1858–68. 22622349 10.1038/ismej.2012.43 PMC3446812

[40] McMurdie PJ, Holmes S Waste not, want not: why rarefying microbiome data is inadmissible. PLoS Comput Biol 2014; 10:e1003531. 24699258 10.1371/journal.pcbi.1003531 PMC3974642

[41] Maccaferri S, Vitali B, Klinder A, Kolida S, Ndagijimana M, Laghi L, Calanni F, Brigidi P, Gibson GR, Costabile A Rifaximin modulates the colonic microbiota of patients with Crohn's disease: an *in vitro* approach using a continuous culture colonic model system. J Antimicrob Chemother 2010; 65:2556–65. 20852272 10.1093/jac/dkq345

[42] Swidsinski A, Loening-Baucke V, Verstraelen H, Osowska S, Doerffel Y Biostructure of fecal microbiota in healthy subjects and patients with chronic idiopathic diarrhea. Gastroenterology 2008; 135:568–79. 18570896 10.1053/j.gastro.2008.04.017

[43] Ringel Y, Maharshak N, Ringel-Kulka T, Wolber EA, Sartor RB, Carroll IM High throughput sequencing reveals distinct microbial populations within the mucosal and luminal niches in healthy individuals. Gut Microbes 2015; 6:173–81. 25915459 10.1080/19490976.2015.1044711 PMC4615648

[44] Fouhy F, Ross RP, Fitzgerald GF, Stanton C, Cotter PD Composition of the early intestinal microbiota: knowledge, knowledge gaps and the use of high-throughput sequencing to address these gaps. Gut Microbes 2012; 3:203–20. 22572829 10.4161/gmic.20169 PMC3427213

[45] Tu Q, He Z, Zhou J Strain/species identification in metagenomes using genome-specific markers. Nucleic Acids Res 2014; 42:e67. 24523352 10.1093/nar/gku138 PMC4005670

