# Supplementary material for: Rifaximin is associated with modest, transient decreases in multiple taxa in the gut microbiota of patients with diarrhoea-predominant irritable bowel syndrome
Source: Gut Microbes. 2018 Jul 18;10(1):22–33. doi: 10.1080/19490976.2018.1460013 (PMC6363070; doi:10.1080/19490976.2018.1460013)
Supplement: 1052206_supplemental_files.ppt [file kgmi-10-01-1460013-s002.docx]

**Supplementary material**

**Supplementary Text**

**Reproducibility of technical replicates**

Issues with reproducibility may impact utilisation of microbial sequence data in a clinical setting. To demonstrate reproducibility, we performed a pilot study of 96 stool samples (n = 23) collected at different time points. Samples were divided into 3 separate aliquots, which underwent independent extraction, polymerase chain reaction (PCR), and sequencing steps. A high degree of reproducibility was observed. For example, measurements of 1 family (Fusobacteria) were highly similar across the 3 technical replicates (**Supplementary Figure S2A-B**). Across all taxa, there was nearly perfect reproducibility for taxa with a relative abundance of 10^-3^ (**Supplementary Figure S2C**). Generally, Pearson correlations between the technical replicates were ≥ 0.8 for families with relative abundances of ≥ 10^-3^ and ≥ 0.6 for relative abundances of ≥ 10^-4^ (**Supplementary Figure S2C**). The average number of sequences per sample in the pilot dataset was ~ 10^6^; thus, it was not surprising to see that loss in reproducibility in rare taxa as the frequency with which taxa were observed approaches the sequencing depth per sample. Nonetheless, the overall high level of reproducibility demonstrated the ability to capture a consistent microbial signature within a sequencing batch across different extraction, PCR, and sequencing reactions for all but the rarest taxa.

**Alternative de novo analysis path with DADA 2**

In order to determine how the results of our paper would have changed under a different analysis path, an additional comparison of the clinic visit (V)3 time points with the V4 and V11 time points using DADA2 was performed.^1^ DADA2 allows for identification of sequence clusters independent of comparison to a reference database. In the current analysis, the “big data” suggested pipeline (<https://benjjneb.github.io/dada2/bigdata.html>), which analyses each sample separately, was followed. For the samples with the greatest sequencing depth for each combination of time point and patient, samples were rarefied to 29,841 (the number of sequences in the sample with the smallest numbers of sequences among the V3, V4, and V11 time points). Rarefication was a requirement for our use of DADA2 because de novo operational taxonomic unit (OTU) clustering algorithms like DADA2 do not easily scale to the millions of sequences that were present in many of our samples. Each sample was filtered and fed independently through DADA2, and the resulting species-level operational taxonomic units (sOTUs) were collapsed across samples at 100% identity. sOTUs that were present in less than 10% of samples were removed. In comparing the average sequence count, a stronger correspondence between the V3 and V11 time points (with an r-squared value of 0.86) was observed, compared with the V3 and V4 time points (with an r-squared of 0.8; **Supplementary Figure S4**), although there are no individual sOTUs that are significantly different between time points at a 10% false discovery rate (data not shown). As one might expect,^2^ there is lower power to resolve individual OTUs under a rarefication scheme than under the relative abundance scheme used in the main paper. Nonetheless, the stronger correspondence between the V3 and V11 time points than the relationship between the V3 and V4 time points is consistent with rifaximin having a small, transient impact on the gut microbial community, with the community recovering from antibiotic exposure over time.

**SUPPLEMENTARY TABLES**

**Supplementary Table S1.** The time points collected for patients and the number of sequences in each sample for the sample with the most sequences.

**Supplementary Table S2.** Differences between the V3 and V4 time points at the genus level. p values are from the Wilcoxon test and were adjusted utilizing the Benjamini-Hochberg FDR method.

FDR: false discovery rate; V: clinic visit.

**Supplementary Table S3.** Results for mixed-linear models visualised in **Supplementary Figure S1**. p values for the Wilcoxon test were generated on non–log-transformed data (simple relative abundance). The columns are as follows:

names: The name of the taxa.

pValuesTime: p values for the null hypothesis that V3 = V4 = V11 (i.e. that there is no difference in the relative abundance by time point). These p values were not corrected for multiple hypothesis testing.

pValuesSubject: p values for the null hypothesis that there is no association of the taxa with subject. These p values were not corrected for multiple hypothesis testing.

pValuesV3_V4: p values for the null hypothesis that V3 is different from V4 from a paired Wilcoxon test. These p values were not corrected for multiple hypothesis testing.

pValuesV3_V11: p values for the null hypothesis that V3 is different from V11 from a paired Wilcoxon test. These p values were not corrected for multiple hypothesis testing.

meanBugV3: The average of all the relative abundance for the taxa at the V3 time point.

meanBugV4: The average of all the relative abundance for the taxa at the V4 time point.

meanBugV11: The average of all the relative abundance for the taxa at the V11 time point.

sdBugV3, sdBugV4, sdBugV11: The standard deviations associated with the means.

isTaxa: False for Shannon diversity, rarified richness, and number of sequences; true for all else.

adjustedTime, adjustedSubject, adjusted_wilcox_V3_V4, adjusted_wilcox_V3_V11. p values were adjusted utilising the Benjamini-Hochberg FDR method.

FDR: false discovery rate; V: clinic visit.

**Supplementary Table S4.** Results of statistical modelling for the paired Wilcoxon test comparing the V3 and V4 time points. For tables S4–S7, statistical tests were run on log-transformed data (using the equation for log transformation supplied in the Methods). “protocolpValue” is the uncorrected p value and “adjustedP” is the Benjamini-Hochberg FDR–corrected p value. R-squared values are from simple linear models.

FDR: false discovery rate; V: clinic visit.

**Supplementary Table S5.** Results of statistical modelling for the paired Wilcoxon test comparing the V6 versus V7 time points. V: clinic visit.

**Supplementary Table S6.** Results of statistical modelling for the paired t-test for the V6 versus V7 time points for rifaximin-treated patients. V: clinic visit.

**Supplementary Table S7.** Results of statistical modelling for the paired t-test for the V6 versus V7 time points for placebo-treated patients. V: clinic visit.

**SUPPLEMENTARY FIGURES**

**Supplementary Figure S1.** For each of the 74 non-rare taxa seen in at least 10% of samples, plus Shannon diversity, rarified richness, and the number of sequences per sample, the relative abundance is shown as a function of time point (top panel) and subject ID (bottom panel). p values were uncorrected for multiple hypothesis testing and were from a mixed linear model evaluating the null hypothesis that the fixed time (V3/baseline vs V4/end_open_label_treatment vs V11/end_of_study) and random patient terms were not associated with the relative abundance. The "icc" is the intra-class correlation coefficient for patient estimating how much variation for each taxon is explained by patient. V: clinic visit.


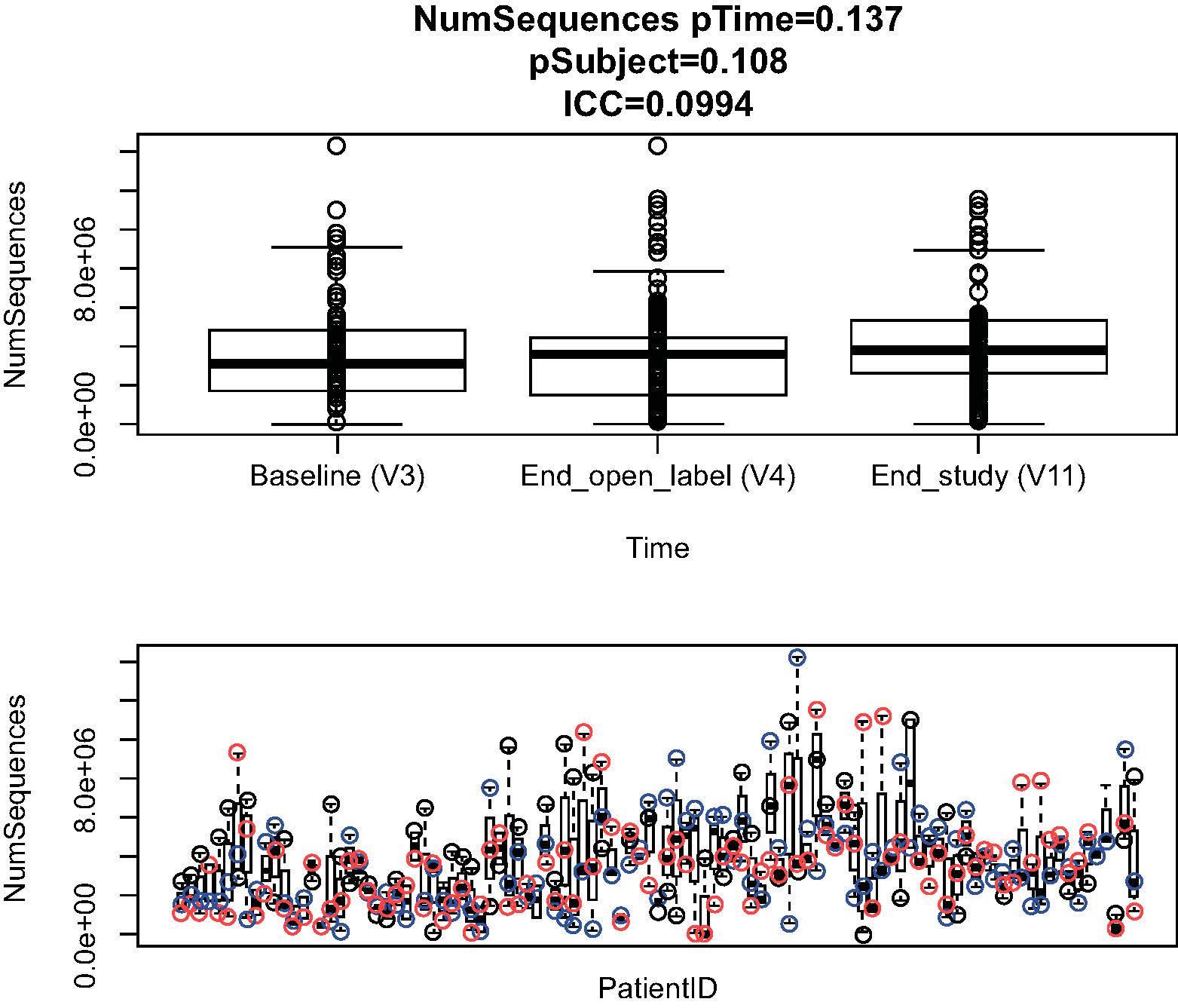


**Supplementary** **Figure S2.** Technical replicates demonstrated a high level of reproducibility across separate extraction, PCR, and sequencing steps. (A–B) For 1 taxon (Fusobacteria), measures of log-normalised relative abundance are shown for the first versus the third (A) and second versus the third (B) technical replicates. (C) For all taxa, the Pearson correlations for the 3 possible comparisons of the 3 technical replicates (different shaded points) are plotted against the relative abundance of each taxon. A small amount of jitter was added to data points in (C) to aid with visualisation.

PCR: polymerase chain reaction; r: correlation coefficient.


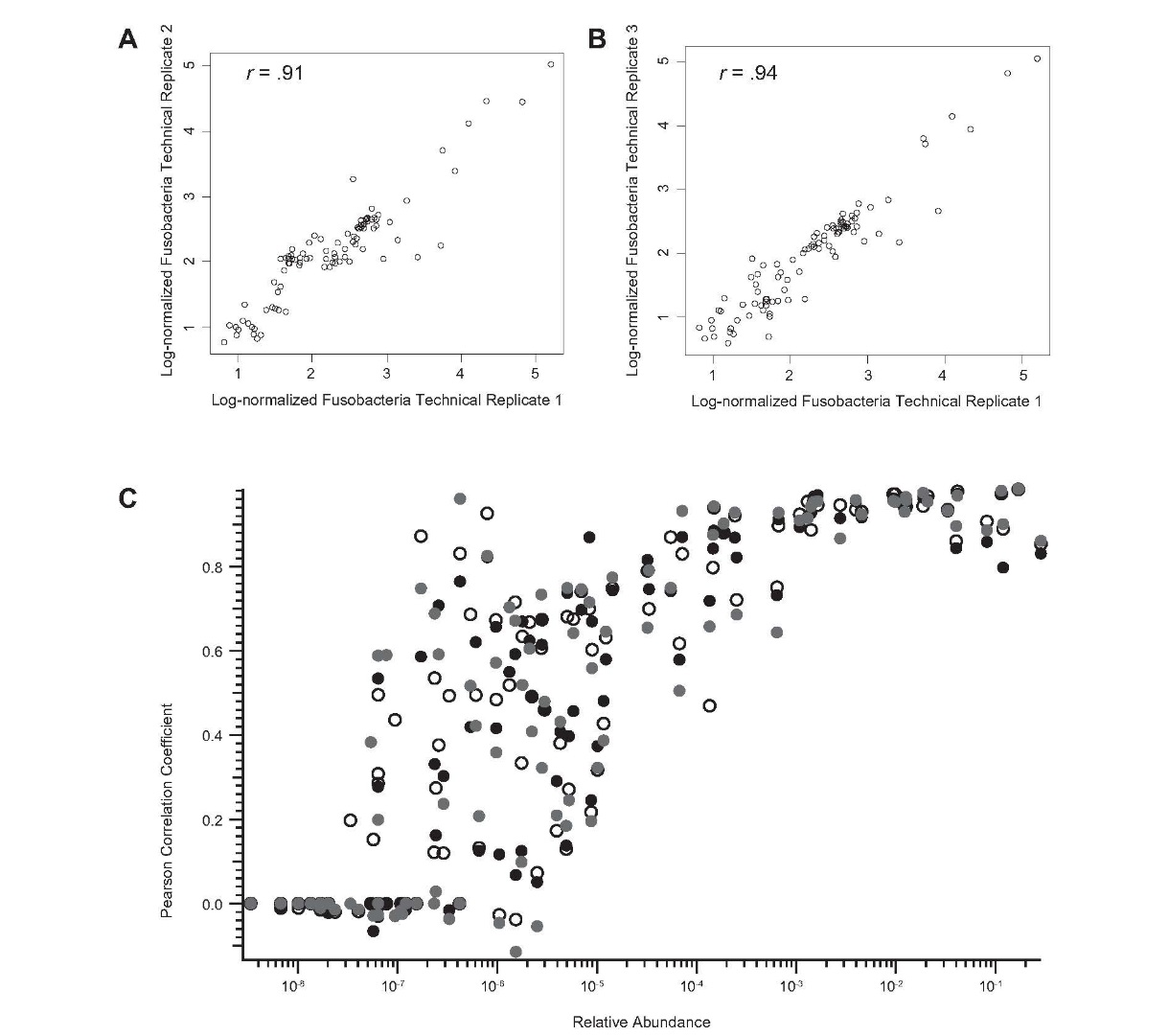


**Supplementary Figure S3.** For each of the 74 non-rare taxa seen in at least 10% of samples at the V3 time point, plus Shannon diversity, rarified richness, and the number of sequences per sample, the relative abundance is shown as a function of responder status. p values were determined by unpaired Wilcoxon test and were not corrected for multiple hypothesis testing.

V: clinic visit.

**
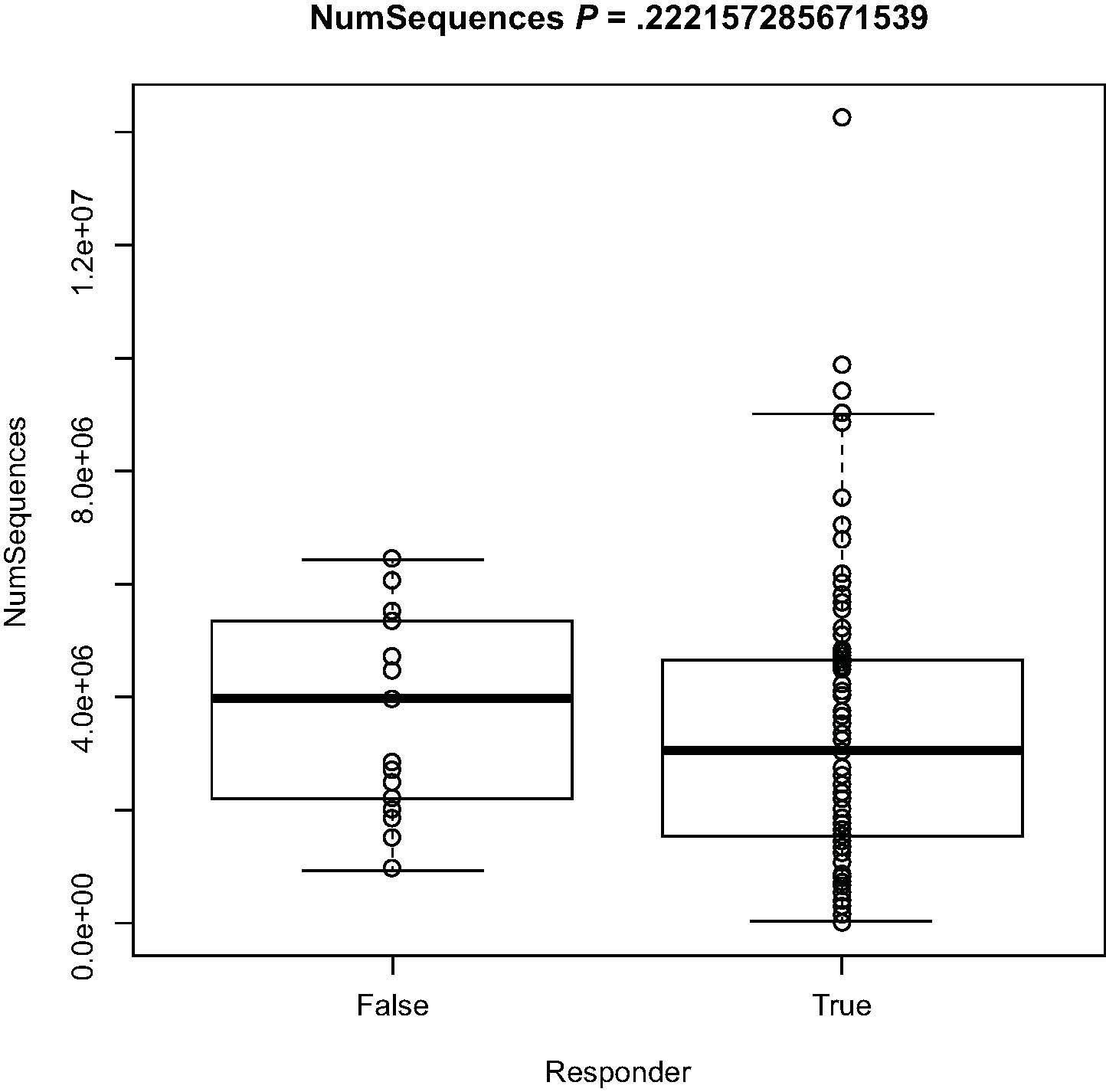
**

**Supplementary Figure S4.** Comparison of mean abundances for sOTUs clustered under DADA2 for V3 vs V4 (A) and V3 vs V11 (B). No significant differences were observed between either set of time points at a 10% false discovery rate.

sOTU: species-level operational taxonomic units; V: clinic visit.


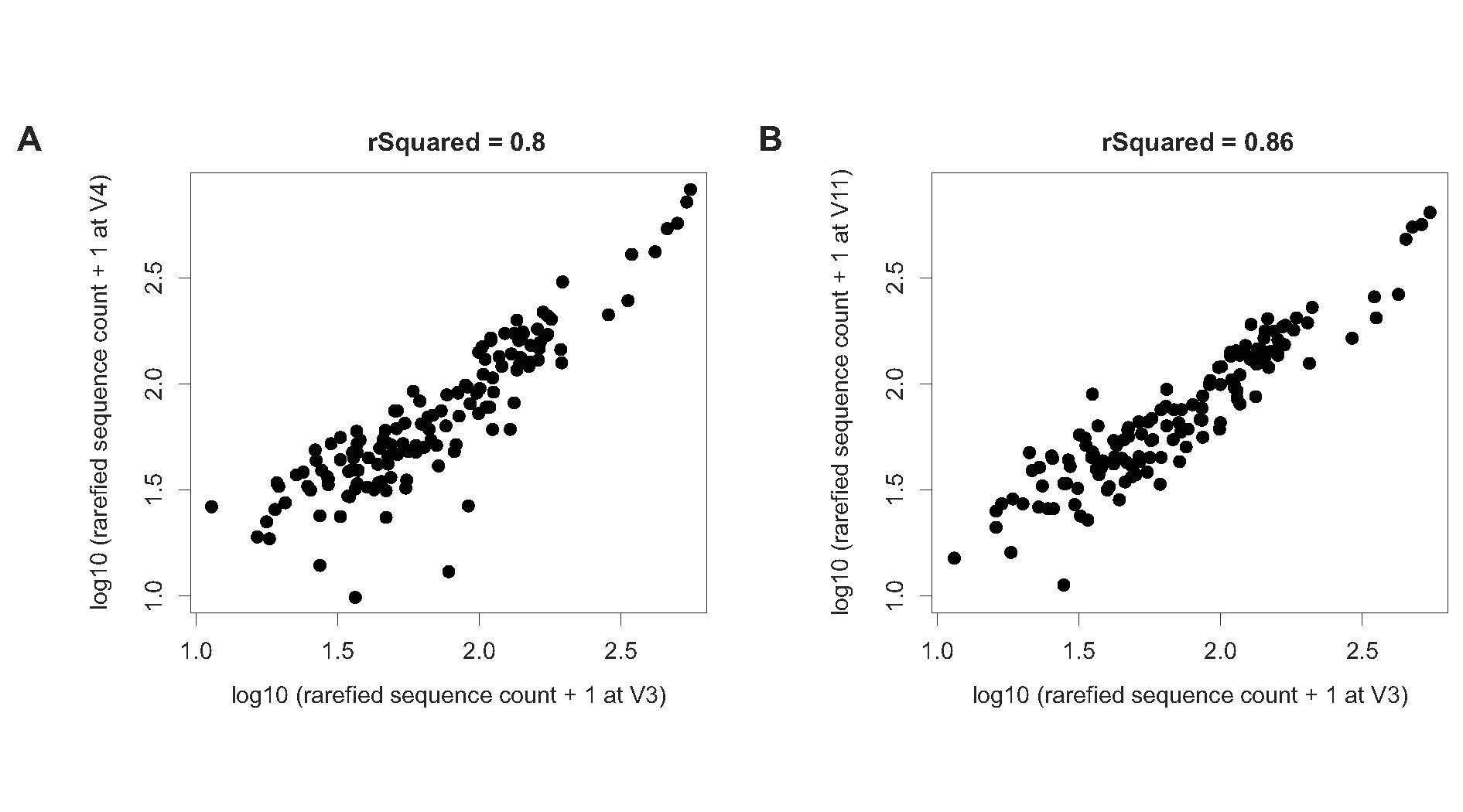


**Supplementary Figure S5.** Comparison of the paired Wilcoxon test comparing batches 1 and 2 vs. batches 3 and 4 (see Figure 2 in the main paper). V: clinic visit.


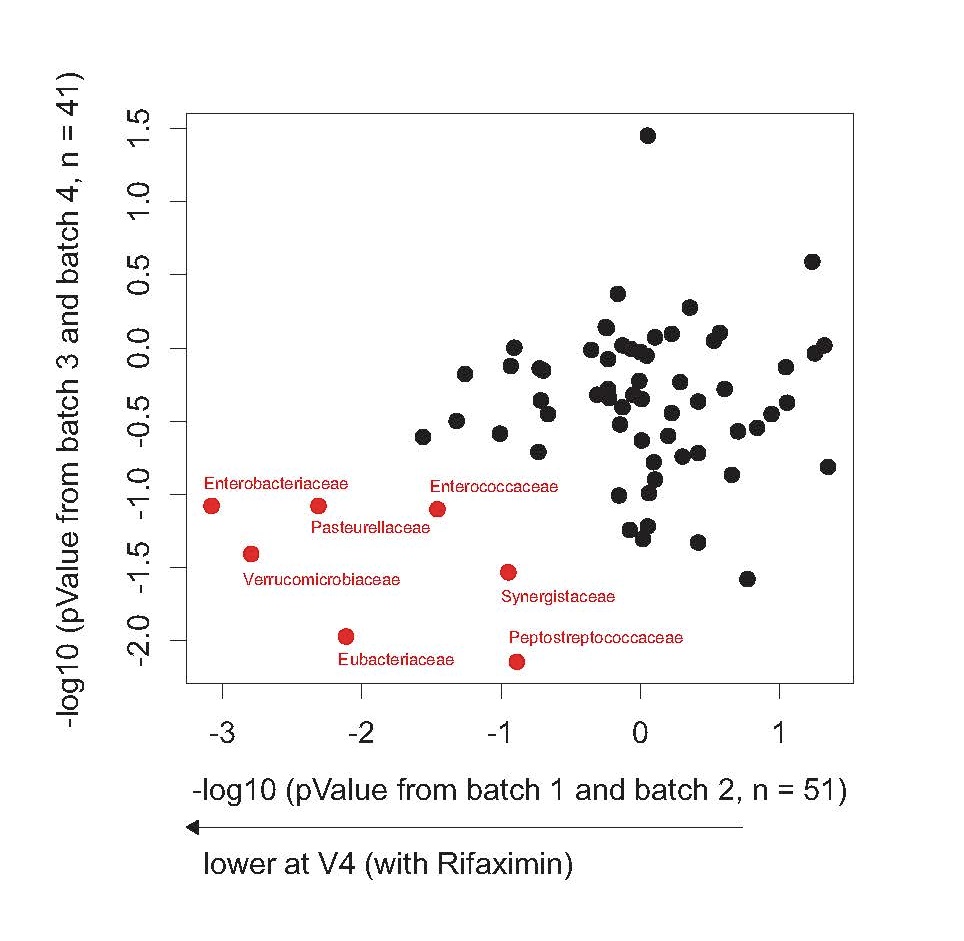


**References**

1. Callahan BJ, McMurdie PJ, Rosen MJ, Han AW, Johnson AJ, Holmes SP. DADA2: High-resolution sample inference from Illumina amplicon data. Nat Methods 2016; 13:581-3.

2. McMurdie PJ, Holmes S. Waste not, want not: why rarefying microbiome data is inadmissible. PLoS Comput Biol 2014; 10:e1003531.
